# A Genome-Wide Search for Linkage of Estimated Glomerular Filtration Rate (eGFR) in the Family Investigation of Nephropathy and Diabetes (FIND)

**DOI:** 10.1371/journal.pone.0081888

**Published:** 2013-12-17

**Authors:** Farook Thameem, Robert P. Igo, Barry I. Freedman, Carl Langefeld, Robert L. Hanson, Jeffrey R. Schelling, Robert C. Elston, Ravindranath Duggirala, Susanne B. Nicholas, Katrina A. B. Goddard, Jasmin Divers, Xiuqing Guo, Eli Ipp, Paul L. Kimmel, Lucy A. Meoni, Vallabh O. Shah, Michael W. Smith, Cheryl A. Winkler, Philip G. Zager, William C. Knowler, Robert G. Nelson, Madeline V. Pahl, Rulan S. Parekh, W. H. Linda Kao, Rebekah S. Rasooly, Sharon G. Adler, Hanna E. Abboud, Sudha K. Iyengar, John R. Sedor, on behalf of the Family Investigation of Nephropathy and Diabetes Research Group

**Affiliations:** 1 Department of Medicine, The University of Texas Health Science Center, San Antonio, Texas, United States of America; 2 Department of Epidemiology and Biostatistics, Case Western Reserve University, Cleveland, Ohio, United States of America; 3 Department of Internal Medicine, Wake Forest School of Medicine, Winston-Salem, North Carolina, United States of America; 4 Phoenix Epidemiology and Clinical Research Branch, National Institute of Diabetes and Digestive and Kidney Diseases, National Institutes of Health, Phoenix, Arizona, United States of America; 5 Department of Medicine, Case Western Reserve University, Cleveland, Ohio, United States of America; 6 Department of Genetics, Texas Biomedical Research Institute, San Antonio, Texas, United States of America; 7 Department of Medicine, University of California, Los Angeles, California, United States of America; 8 Center for Health Research, Kaiser Permanente Northwest, Portland, Oregon, United States of America; 9 Department of Pediatrics, Harbor-University of California Los Angeles Medical Center, Torrance, California, United States of America; 10 Department of Medicine, Harbor-University of California Los Angeles Medical Center, Torrance, California, United States of America; 11 National Institute of Diabetes, Digestive and Kidney Diseases, National Institutes of Health, Bethesda, Maryland, United States of America; 12 Department of Epidemiology and Medicine, Johns Hopkins University, Baltimore, Maryland, United States of America; 13 University of New Mexico, Albuquerque, New Mexico, United States of America; 14 National Human Genome Research Institute, NIH, Bethesda, Maryland, United States of America; 15 Center for Cancer Research, National Cancer Institute, NIH, Frederick, Maryland, United States of America; 16 Department of Medicine, University of California, Irvine, California, United States of America; 17 Department of Pediatrics, University of Toronto, Toronto, Canada; NIDCR/NIH, United States of America

## Abstract

**Objective:**

Estimated glomerular filtration rate (eGFR), a measure of kidney function, is heritable, suggesting that genes influence renal function. Genes that influence eGFR have been identified through genome-wide association studies. However, family-based linkage approaches may identify loci that explain a larger proportion of the heritability. This study used genome-wide linkage and association scans to identify quantitative trait loci (QTL) that influence eGFR.

**Methods:**

Genome-wide linkage and sparse association scans of eGFR were performed in families ascertained by probands with advanced diabetic nephropathy (DN) from the multi-ethnic Family Investigation of Nephropathy and Diabetes (FIND) study. This study included 954 African Americans (AA), 781 American Indians (AI), 614 European Americans (EA) and 1,611 Mexican Americans (MA). A total of 3,960 FIND participants were genotyped for 6,000 single nucleotide polymorphisms (SNPs) using the Illumina Linkage IVb panel. GFR was estimated by the Modification of Diet in Renal Disease (MDRD) formula.

**Results:**

The non-parametric linkage analysis, accounting for the effects of diabetes duration and BMI, identified the strongest evidence for linkage of eGFR on chromosome 20q11 (log of the odds [LOD] = 3.34; *P* = 4.4×10^−5^) in MA and chromosome 15q12 (LOD = 2.84; *P* = 1.5×10^−4^) in EA. In all subjects, the strongest linkage signal for eGFR was detected on chromosome 10p12 (*P* = 5.5×10^−4^) at 44 cM near marker rs1339048. A subsequent association scan in both ancestry-specific groups and the entire population identified several SNPs significantly associated with eGFR across the genome.

**Conclusion:**

The present study describes the localization of QTL influencing eGFR on 20q11 in MA, 15q21 in EA and 10p12 in the combined ethnic groups participating in the FIND study. Identification of causal genes/variants influencing eGFR, within these linkage and association loci, will open new avenues for functional analyses and development of novel diagnostic markers for DN.

## Introduction

Diabetes mellitus is responsible for approximately 50% of cases of incident end-stage renal disease (ESRD) in the United States and other Western societies, with projections of up to 70% of ESRD in 2015 [1]. Diabetic nephropathy (DN) is a serious complication of diabetes caused by hyperglycemia-induced renal injury, involving a complex interplay of metabolic and hemodynamic disturbances in genetically predisposed individuals. DN is typically characterized by persistent proteinuria and elevated blood pressure; however, progressive declines in estimated glomerular filtration rate (eGFR, an estimate of kidney function) are uniformly present and may occur in the absence of persistent proteinuria [2]. Individuals with DN have significantly increased cardiovascular morbidity and premature mortality. Among Pima Indians with type 2 diabetes, only those with overt DN had mortality rates higher than among nondiabetic persons [3]. Rates of decline in eGFR were associated with albuminuria in type 2 diabetes [4] and assessments of eGFR facilitate the diagnosis, evaluation and management of patients with chronic kidney disease. Therefore, identifying the inherited and environmental causes of reduced eGFR would help target novel treatment strategies to prevent progression of DN to ESRD and reduce associated cardiovascular complications.

Epidemiological studies demonstrate that eGFR is a complex trait, whose level in a given individual reflects contributions from genes whose expression is modulated by a hyperglycemic environment. Genome-wide linkage and association analyses have been used to localize susceptibility genes influencing eGFR. Several prior genome-wide linkage scans, including our previous genome scan in a subset of the same study subjects, identified positional candidate genes potentially influencing eGFR based on implicated chromosomal regions [5]–[15]. Recently, genome-wide association studies (GWAS) have localized common variants influencing eGFR [16]–[24]. However, these common variants account for a modest genetic contribution to variation in eGFR and related traits and their functional significance remains to be elucidated.

In an attempt to identify and characterize susceptibility genes influencing kidney disease in diabetes, we chose the family-based genome-wide linkage scan approach that can identify genetic regions where there are multiple susceptibility variants or other complex mechanisms that may in aggregate explain a larger proportion of the heritability than the single polymorphisms typically identified in GWAS. A genome-wide linkage screen was performed for eGFR based on 6,000 single nucleotide polymorphisms (SNPs) from Hispanic American (HA), African American (AA), European American (EA), and American Indian (AI) participants in the Family Investigation of Nephropathy and Diabetes (FIND). The FIND study was established to provide genome-wide coverage for localization of genes with pathogenically significant effects on risk of progressive DN and related traits, such as eGFR.

## Materials and Methods

### Study Participants

The FIND study protocol and patient recruitment procedures have been reported [25]. Briefly, families of self-reported AA, EA, AI and MA ethnicity were recruited from eight participating investigation centers. Families were ascertained based on a proband with advanced diabetic nephropathy (DN) or DN-attributed end-stage renal disease (ESRD), who had at least one additional diabetic sibling with or without DN. A variety of metabolic, hemodynamic, anthropometric, and demographic variables were collected. Diabetes was clinically diagnosed based on treatment regimen (insulin or oral hypoglycemic agents); the remainder of study participants were screened using hemoglobin A1C levels or fasting plasma glucose concentrations. Details of the proband and sibling selection criteria have been described [14]. The Institutional Review Board at each participating center (Case Western Reserve University, Cleveland, OH, Harbor-University of California Los Angeles Medical Center, Johns Hopkins University, Baltimore, National Institute of Diabetes and Digestive and Kidney Diseases, Phoenix, AZ, University of California, Los Angeles, CA, University of New Mexico, Albuquerque, NM, University of Texas Health Science Center at San Antonio, San Antonio, TX, Wake Forest School of Medicine, Winston-Salem, NC) approved all procedures, and all study subjects provided written informed consent. A certificate of confidentiality was filed at the National Institutes of Health.

### Estimation of GFR

**e**GFR was estimated using the Modification of Diet in Renal Disease (MDRD) equation (Levey et al., 1999): eGFR (ml/min per 1.73 m^2^) = 186×(plasma creatinine)^−1.154^×(age)^−0.203^×(0.742 if female)×(1.210 if AA). For patients with ESRD (N = 1275) receiving dialysis treatments or kidney transplants, eGFR was imputed at 5.0 ml/min/1.73 m^2^ because (1) eGFR is meaningless with respect to the participant's true kidney function under these circumstances; and (2) imputing at zero, an extreme value, would give the data from ESRD cases undue influence relative to those of the non-ESRD cases. A total of 3960 subjects, comprising 3547 sib pairs, were included in the analysis (Table 1).

**Table 1:** Summary of analyzed pedigrees and genotyped individuals (N).

Ethnic Group	Pedigrees	Individuals	Full-Sib Pairs	Half-Sib Pairs
African American	346	954	705	149
American Indian	212	781	708	147
European American	199	614	662	26
Mexican American	478	1611	1472	120
**Total**	**1235**	**3960**	**3547**	**442**

### Genotyping

DNA was isolated from lymphoblastoid cell lines or leukocyte buffy coats [25]. The Illumina SNP-based Linkage Panel IVb was employed for both linkage and association analysis as described previously [26]. This panel consists of 6,008 diallelic SNP markers distributed evenly across the genome. The average and median intervals between markers are 482 kb (0.64 cM) and 298 kb (0.35 cM), respectively. The largest interval between successfully genotyped markers is 5.02 cM on chromosome 8, and linkage disequilibrium (LD) between markers is minimal. The mean minor allele frequency (MAF) and heterozygosity of SNP markers are 37% and 44%, respectively. Genotyping was performed at the Center for Inherited Disease Research (CIDR).

### Statistical methods

Genetic analyses were performed using the S.A.G.E. (Statistical Analysis for Genetic Epidemiology) software package, version 5.3 (http://darwin.cwru.edu/sage/). Allele frequencies were estimated separately in the four ethnic groups using the maximum likelihood method implemented in the program FREQ. Mendelian inconsistencies were identified with the MARKERINFO program and inconsistent genotypes were coded as missing. Errors in relationship specification were identified with the program RELTEST. When necessary, a second relationship testing program, RELPAIR version 2.0.1, was enlisted to resolve potential errors involving complex relationships. Multipoint identity by descent (IBD) allele sharing probabilities were estimated by the method of maximum likelihood, using all available information in the pedigree as implemented in the program GENIBD. Multipoint IBD-sharing estimates are robust to misspecification of population allele frequencies, as may occur with admixed samples, because most of the parental information is inferred when the available information is high [27]. The Shannon information, as calculated by Merlin [28], available from the Illumina IV SNP panel was never less than 0.7, and seldom less than 0.8, except at the telomeric regions (data not shown). Using the multipoint IBD sharing estimates, a genome-wide linkage scan for quantitative trait loci potentially influencing eGFR was performed by the Haseman-Elston regression approach implemented within the program SIBPAL, using the W4 weighting option to maximize power. We converted the *p* values reported by SIBPAL to LOD scores using the one-sided chi-squared distribution with one degree of freedom (i.e., a 50∶50 mixture of distributions with 0 and 1 degrees of freedom), appropriate for a one-sided test. In principle, the sib pairs who are identical by descent (IBD) at a marker locus will be phenotypically similar for traits influenced by a nearby linked gene. Evidence for linkage of eGFR was assessed with and without incorporating covariate effects of diabetes duration and body mass index (BMI), entered in the regression model as the sibpair sum. Non-parametric multipoint linkage analysis was carried out separately in each ethnic group, and *P* values were combined across ethnicities according to Fisher's method [29]. Empirical *P* values were obtained for the major linkage peaks using the “simulation” option in SIBPAL, which performs a permutation test. Association analysis was conducted as described previously [26] using the linear mixed model approach implemented in the S.A.G.E. program ASSOC. Results were combined across ethnic groups using Fisher's method [26], [29]. The SNPs used in this analysis have been previously reported [26]. To assess the sensitivity of the association analysis to genetic admixture, the linear mixed model was fitted with and without adjustment for the first two principal components from a principal components analysis using 5,547 SNPs from the Illumina IV panel with minor allele frequencies of at least 0.05 in the combined sample. Principal components were obtained via the smartpca program in EIGENSOFT [30].

## Results

Several quality control measures were implemented to determine the final set of markers for the linkage analysis. Briefly, SNPs were required to have median GenCall scores (a measure of how close a genotype is to the center of the cluster of other samples assigned to the same genotypes) ≥0.5, MAF (specific to ethnic group) ≥0.05, and *p* value for deviation from Hardy-Weinberg proportions >0.001. Since, LD between neighboring SNPs may create bias in estimates of IBD sharing among relatives, markers were screened such that pairwise | D′ | was less than 0.3. After quality control, a final marker set of SNPs qualifying for further genetic analysis was identified as described previously [24]. Table 1 lists the ethnicities of the 3,960 subjects comprising 3,547 sib pairs and 442 half-sib pairs from four ethnic groups in whom eGFR and genotypic data were available. Of these, 40.7%, 24.1%, 19.7%, and 15.5% were MA, AA, AI, and EA, respectively. Table 2 displays the clinical characteristics of genotyped individuals from each ethnic group.

**Table 2:** Clinical characteristics of the genotyped individuals.

Parameters	African American	European American	American Indian	Mexican American
*N*	954	614	781	1611
Male	324 (33.9%)	276 (44.8%)	298 (38.2%)	686 (42.6%)
Age (years)	59 (51, 67)	62 (53, 70)	53 (46, 61)	57 (49, 65)
Diabetes duration (years)	18 (11, 25)	18 (11, 26)	16 (4, 25)	15 (7, 22)
BMI (kg/m^2^)	32 (27, 37)	30 (26, 36)	32 (27, 37)	30 (26, 34)
HbA1c (%)	7.1 (6.2, 8.6)	7.0 (6.2, 8.0)	7.5 (6.3, 9.3)	7.5 (6.4, 9.1)
Urine ACR (g/g)	0.15 (0.01, 3.00)	0.05 (0.01, 1.33)	0.13 (0.02, 3.00)	0.05 (0.01, 1.53)
Urine PCR (g/g)	0.31 (0.06, 3.50)	0.21 (0.06, 2.00)	0.37 (0.09, 3.50)	0.18 (0.05, 2.20)
eGFR (ml/min per 1.73 m^2^)	46.6 (5.0, 83.0)	52.2 (5.0, 77.9)	60.5 (5.0, 97.4)	69.1 (5.0, 100.3)

ACR-Albumin:Creatinine Ratio; PCR-Protein:Creatinine Ratio; eGFR-estimated Glomerular Filtration Rate using modified MDRD equation Values are expressed as either N (%) or median (1st quartile, 3rd quartile).

### Genome-wide linkage scans for eGFR

**Figure 1:**
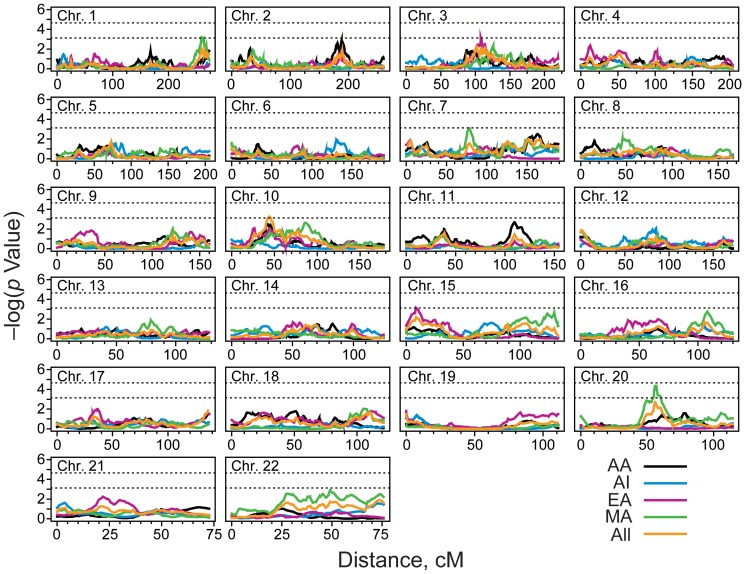
This figure shows the results of the genome-wide linkage scan for eGFR in population-specific and population-combined analysis that accounted the covariate effects of BMI and diabetes duration. AA-African Americans; AI-American Indians; EA-European Americans; MA-Mexican Americans, cM-Centi Morgans.

**Figure 2:**
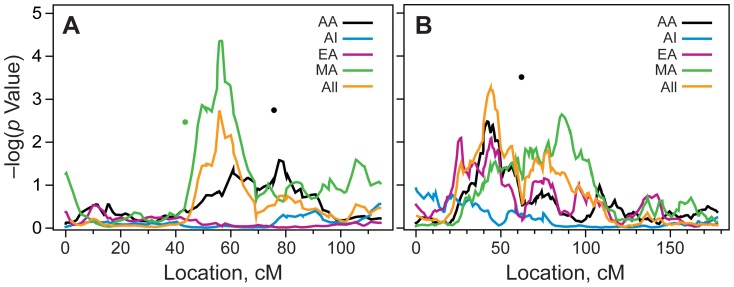
Figure 2a. The figure shows the linkage of eGFR on 10p12 after accounting for the covariate effects of BMI and diabetes duration in the population-specific and combined analysis. A black dot denotes the location of rs1345561 (62.24 cM) that is associated with eGFR in African American participants (P  =  3.1 × 10^−4^) after accounting for the covariate effects of BMI and diabetes duration. AA-African Americans; AI-American Indians; EA-European Americans; MA-Mexican Americans, cM-Centi Morgans. Figure 2b.The figure shows the linkage of eGFR on 20q11 after accounting for the covariate effects of BMI and diabetes duration in the population-specific and combined analysis. The green and black dots denote the location of rs1885567 (43.47 cM) rs968478 (75.75 cM) that are associated with eGFR in MA (P  =  0034) and AA (P = 0.0018) respectively. AA-African Americans; AI-American Indians; EA-European Americans; MA-Mexican Americans; cM-Centi Morgans.

Adjusting for the covariate effects of diabetes duration and BMI, the genome-wide linkage scan in population-combined data identified the strongest evidence for linkage of eGFR on chromosome 10p12.31 (*P* = 5.5×10^−4^) at 44 cM near rs1339048 (Figures 1, 2a and Table 3). Evidence for linkage was primarily contributed by the AA and EA groups, with a smaller contribution from MA. A second suggestive linkage signal across populations was observed on chromosome 20q11 at 56 cM (P = 1.9×10^−3^), flanked by SNPs rs221972 and rs735264 (Figure 1; Table 3).

**Table 3:** Major linkage peaks identified for the eGFR in population-specific and in population-combined analysis.

Chr.	Group	cM	Marker	*p*, Baseline	LOD, Baseline	*p*, Covariates	LOD, Covariates	Flanking Markers
1	MA	259.4	rs1537802	4.7×10^−4^	2.38	6.2×10^−4^	2.26	rs1341446, rs1342872
2	EA	183.7	rs963854	6.0×10^−3^	1.37	3.9×10^−2^	0.67	rs2007326, rs1002207
	AA	187.8	rs767042	1.1×10^−3^	2.04	1.5×10^−3^	1.91	rs8899060, rs2012128
3	EA	107.8	rs1562626	1.4×10^−2^	1.05	7.5×10^−4^	2.19	rs1470797, rs1494302
7	MA	78.4	rs1468588	1.0×10^−3^	2.07	7.5×10^−4^	2.19	rs1874243, rs678798
10	EA	26.6	rs913375	1.9×10^−4^	2.74	8.1×10^−4^	2.16	rs1033912, rs1535976
	All	43.9	rs1339048	1.2×10^−3^	—	5.5×10^−4^	—	rs7292450, rs949857
15	EA	11.1	rs2928714	1.2×10^−3^	2.00	1.5×10^−4^	2.84	rs3922665, rs1862359
20	MA	56.8	rs736264	3.5×10^−5^	3.43	4.4×10^−5^	3.34	rs2219720, rs663550
	All	56.0	—	2.2×10^−3^	—	1.9×10^−3^	—	rs2219720, rs735264

Group, ethnic group (AA = African American, AI = American Indian, EA = European American, MA = Mexican American); cM, centimorgans on the deCODE linkage map; Baseline, eGFR without covariate adjustment; Covariates, eGFR adjusted for BMI and diabetes duration. Reported *p* values are asymptotic.

In population-specific analysis, the strongest evidence for linkage of eGFR localized to a genetic region on 20q11.22 at 56.8 cM near rs736264 (LOD = 3.34; *P* = 4.4×10^−5^) in MA participants (Figures 1, 2b). Other genetic regions with suggestive evidence for eGFR linkage in MA included 1q43 and 7q11.22 (Figure 1; Table 3). Accounting for the covariate effects of diabetes duration and BMI, the Haseman-Elston linkage scan in EA identified the strongest linkage signal for eGFR on chromosomes 15q12 near rs2928714 (LOD = 2.84; *P* = 1.5×10^−4^) and suggestive evidence for linkage was found near rs913375 on 10p14 (Figure 1; Table 3).

### Genome-wide association scans for eGFR

Following the linkage scans, coarse association analyses between eGFR and SNPs that passed quality control was performed, using the approach implemented in ASSOC. Table 4 shows the SNPs associated with eGFR with *p*<0.001 in at least one ethnic group after adjusting for the covariate effects of diabetes duration and BMI in population-specific or population-combined analyses. In the population-combined data, the most significant association with eGFR was found for rs486567 on chromosome 1q21.1 (P = 2.9×10^−4^), primarily contributed by EA (Table 4). We also observed a significant association between rs580839 residing on 15q14 and eGFR (P = 4.2×10^−4^) in the combined data, that was primarily driven by AI (Table 4). Our association analysis in the population combined data also exhibited a significant association between rs856830 residing on 6q12 and eGFR (P = 4.3×10^−4^), which was driven by AA, AI and MA. Another SNP (rs1345561) associated with eGFR (P = 6.4×10^−4^) in the combined data was located approximately 16 Mb from the eGFR linkage marker rs1339048 on 10p12 (Table 4; Figure 2). This association in the combined data was primarily driven by AA (Table 4). Several SNPs were significantly associated with eGFR in population-specific analyses, the most significant were rs1703711 residing on chromosome 10q26.3 (P = 2.96×10^−4^), rs580839 on 15q14 (P = 4.81×10^−5^), rs666478 on 9p21 (P = 1.46×10^−4^) and rs2928972 on 18q21.2 (P = 1.30×10^−4^) in AA, AI, EA, and MA, respectively (Table 4). These results were robust to ethnic admixture: no *P* value changed by more than 2-fold, and the vast majority of *P* values changed by less than 1.1-fold, with adjustment for two principal components from a genomewide principal components analysis (data not shown).

**Table 4:** Most significantly associated SNPs with eGFR in population-specific and in population-combined analysis.

Chrom	SNP	Position	cM	Population-Specific				Population- Combined
				AA	AI	EA	MA	
				P values*				
1	rs486567	101340030	122.63	0.180395	NA	0.0003104	0.053986	0.00029
1	rs767707	164292200	169.23	0.000914	0.052317	0.4105459	0.930439	0.00526
2	rs1686430	10898399	27.83	0.000788	0.435320	0.4604537	0.967860	0.02468
2	rs1734449	10900882	27.83	0.000698	0.447258	0.4185945	0.997008	0.02205
4	rs925470	25692255	44.72	0.000387	0.229651	0.5795743	0.224038	0.00372
4	rs1965907	88795355	95.34	0.930439	0.000688	0.6808046	0.367687	0.02550
4	rs1516822	153687906	146.15	0.417157	0.000396	0.4176866	0.413709	0.00734
5	rs878953	163859070	168.79	0.537483	0.012537	0.1926391	0.001583	0.00097
5	rs6879805	173474434	191.16	0.724179	0.821073	0.0006116	0.292965	0.01913
6	rs74111	15894581	34.88	0.412605	0.186792	0.9342005	0.000965	0.01408
6	rs6901750	17127405	38.03	0.894238	0.508591	0.0003841	0.212787	0.00892
6	rs2180419	21836296	43.94	0.338171	0.371059	0.8596955	0.000275	0.00755
6	rs856830	68340078	83.08	0.007900	0.032100	0.2815536	0.010168	0.00043
8	rs1904899	21500396	37.4	0.000428	0.646785	0.6082400	0.020144	0.00144
8	rs1019603	113816992	116.33	0.217485	0.000824	0.2168922	0.766197	0.00758
9	rs666478	27163180	49.59	0.519231	0.205288	0.0001459	0.945844	0.00447
9	rs7037744	89983269	94.08	0.028385	0.004670	0.1016464	0.053514	0.00042
10	rs1345561	35810628	62.24	0.000309	0.179033	0.0664067	0.330881	0.00064
10	rs1703711	132531704	171.59	0.000296	0.471069	0.8648250	0.309865	0.00896
11	rs1491846	27855590	44.56	0.822556	0.444531	0.9869061	0.000191	0.01400
12	rs1420725	2621844	5.6	0.797347	6.86E-05	0.5505900	0.338156	0.00338
14	rs762063	52168652	49.94	0.950209	0.250265	0.0005824	0.929830	0.02188
15	rs580839	32786121	31.86	0.271591	4.81E-05	0.5348190	0.102567	0.00042
15	rs11457	61673432	65.78	0.014621	0.000565	0.7720784	0.444003	0.00125
18	rs2928927	47677183	73.73	0.696773	0.881154	0.1955681	0.000129	0.00464
18	rs906507	70889709	108.45	0.651700	0.352213	0.0005717	0.236523	0.00781
19	rs1715093	3505783	12.18	0.000449	0.361667	0.6797823	0.487933	0.01171

SNPs with at least one *p* value less than 0.001 in an ethnic-specific or in the overall analysis are shown. Chrom, Chromosome; SNP, Single Nucleotide Polymorphism; cM, centimorgans; AA, African American; AI, American Indian; EA, European American; MA, Mexican American; NA, Not applicable;

*, Adjusted for the covariate effects of BMI and diabetes duration.

## Discussion

Estimated GFR provides an accurate index of the degree of renal dysfunction and plays a prominent role in the staging of chronic kidney disease [31]. Though variation in eGFR among individuals is partly explained by environmental influences, heritability estimates of eGFR in families suggest that genes play a major role in determining kidney function [32]. Despite high heritability estimates, the identification of genes influencing eGFR and its variability remains challenging. In attempts to identify quantitative trait loci influencing eGFR, the genome-wide linkage approach has been utilized in several genetic epidemiological studies [32]. Genome wide linkage studies have identified several QTL influencing eGFR, but the subsequent susceptibility gene mapping efforts have been unsuccessful and remain in progress. In an effort to identify and characterize the genes influencing kidney function, we performed a SNP-based genome-wide linkage scan followed by association analysis in the multi-ethnic FIND samples.

The most significant linkage to eGFR in ethnicity-combined data was found near rs1339048 on 10p12.31 (P = 5.5×10^−4^). It is interesting to note that the linkage of eGFR on 10p12 was contributed by three (AA, EA, and MA) of the four ethnic groups participating in this study, indicating that the 10p12 region may potentially harbor genes influencing GFR in the FIND participants (Figures 1 and 2a; Table 3). The localization of an eGFR linkage signal in the 10p12 region appears to be novel as this region was not identified in the previous genome-wide microsatellite scan studies for eGFR, including in the FIND [14]. However, several genome-wide microsatellite linkage scans have linked this 10p12 region with obesity and related traits. Furthermore, genes located near the eGFR linkage markers on 10p12, including calcium channel, voltage-dependent, beta 2 subunit (*CACNB2*), ARL5B ADP-ribosylation factor-like 5B (*ARL5*), and nebulette (*NEBL*), were previously associated with eGFR-related traits such as blood pressure and hypertension [33], sudden cardiac arrest and diabetic retinopathy [34]. By binding to actin and thin filaments and Z-line associated proteins in striated cardiac muscle, nubulette regulates cardiac myofibril assembly. CACNB2 is a subunit of a voltage-dependent calcium channel protein and mutations in *CACNB2* were also associated with sudden cardiac arrest.

In population-specific linkage analyses, suggestive evidence for linkage of eGFR was seen at rs736264 on 20q11.22 in MA (Fig. 2b) and at rs2928714 on 15q12 in EA. Although our results failed to replicate genetic regions previously linked to eGFR and related traits in the FIND and non-FIND studies and appeared to detect novel loci influencing eGFR, the 15p12 region has been previously associated with urine albumin∶creatinine ratio (ACR) in MA in the San Antonio Family Diabetes Study [35].

We next performed a sparse association scan to identify whether the SNPs used in the linkage scan are associated with eGFR and potentially responsible for the observed linkage signals. While several SNPs across the genome were suggestively associated with eGFR, none of them were located within the eGFR linkage intervals identified in population-specific or the combined data set. In the population-combined association analysis, the most significant association was observed between eGFR and rs486567, rs580839, and rs1345561 with primary contributors EA, AI, and AA, respectively (Table 4). The rs1345561 SNP is located ∼16 Mb from the linkage of eGFR marker rs1339048 on 10p12 that was primarily driven by AA (Figure 2a; Table 4). The most significant population-specific associations with eGFR were found for rs1703711, rs580839, rs666478, and rs2928972 in AA, AI, EA, and MA, respectively (Table 4).

Of the SNPs most strongly associated with eGFR in population-specific analyses, rs666478 is located within an intronic region of the tyrosine kinase receptor (*TEK*) gene on 9p21. TEK is a cell-surface receptor for angiopoietin (ANGPT) 1, 2, and 4. Through TEK-dependent signaling, ANGPT regulates endothelial cell survival, proliferation, migration, adhesion and cell spreading, and controls vascular permeability and quiescence. Mutations in TEK were previously associated with autosomal dominant forms of venous malformations [36]. Although the functional relevance of rs666478 associating with eGFR needs to be explored, genetic variants located about 5 Mb upstream of *TEK* on cyclin-dependent kinase inhibitor (*CDKN*) 2A, 2B genes have been previously associated with type 2 diabetes mellitus [37] and coronary heart disease [38].

Population-specific association analysis identified several SNPs (rs1686430 and rs1734449) that are associated with GFR only in the AA group. They were located 100 kb apart within an intronic region of the protein disulfide isomerase family A, member 6 (*PDIA6*) gene on 2p25. PDIA6 belongs to a thioredoxin superfamily oxidoreductase from the endoplasmic reticulum that acts as a redox signaling adaptor protein, adjusting reactive oxygen species intermediates to specific signals and redox signals to cell homeostasis [39]. It also catalyzes the formation and isomerization of disulfide bonds thereby facilitating protein folding. Although the functional mechanism by which these two variants residing within the *PDIA6* and regulating renal function needs to be examined, genetic variants located about 7 Mb upstream of *PDIA6* on the SRY (sex determining region Y)-box 11 (*SOX11*) gene were previously associated with T2DM and CKD in Europeans [17].

Utilizing a relatively dense set of 6,000 SNPs as a linkage panel as opposed to the conventional use of a set of about 400 microsatellite markers, the present study reveals quantitative trait loci influencing eGFR to 20q11 in MA, 15q21 in EA and 10p12 in the combined ethnic groups from the FIND study. Several suggestive linkage peaks were also identified in population-specific and population-combined linkage scans in this multi-ethnic cohort. In contrast to GWAS that requires a very stringent p-values (e.g., P<5×10^−8^) for statistical significance on account of the large number of statistical tests involved, linkage studies with less stringent P values are powerful because the number of effectively independent comparisons is much smaller. Conventionally p<0.0001 (LOD>3) has been considered significant linkage, while p<0.001 (LOD>2) has been considered suggestive [40]. Furthermore, the linkage approach can identify potential genetic regions harboring multiple susceptibility variants or other complex mechanisms that may in aggregate explain a larger proportion of the heritability than the single polymorphisms typically identified in GWAS.

As expected for a complex trait, multiple linkage peaks for eGFR were observed. Although the functional relevance of the linkage findings remains to be established and replicated, genetic regions suggestively linked with eGFR in population-specific and population-combined studies suggest that multiple loci are involved in regulating eGFR in diabetes. Disappointingly, there was no significant overlap with loci linked with renal function-related traits in other studies [5]–[13], as well as in our previous FIND microsatellite marker linkage study that was carried out in a subset of the same study populations [14]. Absence of concordance in localizing QTL influencing eGFR between the present study and our previous study [14] using the same FIND population data set could be due to the differences in the sample size, set of linkage markers and covariates used. In contrast to our previous linkage scan for eGFR [14] that used the genotypic data of about 400 microsatellite markers and eGFR data available on 941 individuals and 882 sib pairs, the present study used genotypic data of about 6000 SNPs and eGFR data available on 3960 individuals and 3547 sib pairs. In addition, the previous study accounted for the diabetes duration and angiotensin converting enzyme inhibitor/angiotensin receptor blocker use as covariates in the linkage analysis [14]. The present study used the effects of BMI, and diabetes duration in the eGFR linkage scan. A limitation of the present analysis of eGFR as a continuous variable is that many of the determinants of high eGFR, such as uncontrolled hyperglycemia before diabetes treatment is optimized, may not be under genetic control or may be influenced by different genetic factors than those contributing to declining eGFR. This might, in part, account for differences in the present linkage results with those from analysis of diabetic nephropathy as a discrete trait [30]. The discrepancies between the present study and the non-FIND study results [5]–[13] may be related, in part, to heterogeneous study populations (some with and some lacking diabetes), pedigree structures, ascertainment criteria, treatment effects, definitions of kidney function, and diabetes duration. In contrast to existing publications, the FIND is a multi-ethnic collection of families ascertained based on a proband with advanced DN or ESRD with at least one other diabetic sibling with or without nephropathy. Furthermore, differences in allele frequencies and LD structure of the sets of SNPs contributing to linkage and association might have contributed to the lack of consistency across ethnic groups.

While this large study in a severely affected study sample had several advantages, potential limitations are that eGFR was estimated using a single random blood sample for serum creatinine concentration and employed the modified MDRD equation. This equation performs best for eGFR <60 ml/min per 1.73 m^2^; whereas the CKD-EPI equation appears more accurate for those with eGFR values between 60 and 90 ml/min per 1.73 m^2^. Although all analyses adjusted for diabetes duration and BMI, other potentially relevant confounding variables such as degree of blood pressure control and cardiovascular disease risk factors were unavailable.

In conclusion, several loci influencing eGFR were identified in the multi-ethnic FIND cohort. Linkage and association results emanating from this multi-ethnic study represent a first step towards improving our knowledge of the mechanisms underlying genetic susceptibility to renal function in diabetes. Furthermore, the results of linkage and association analyses reported in this study will help interpret future genome-wide association/whole-genome sequencing data that should accelerate the identification of causal genes for variation in kidney function in patients with diabetes. Defining the genetic architecture responsible for eGFR loss in individuals of different ethnicities may help develop ethnicity-specific intervention programs and services specifically targeted toward this devastating complication of diabetes. With existing high-throughput genome technologies and novel statistical methodologies, we envision promising new therapies to prevent loss of eGFR, a strong and independent risk factor for cardiovascular morbidity and mortality in patients with diabetes.
